# Sleep-Wake Cycling and Energy Conservation: Role of Hypocretin and the Lateral Hypothalamus in Dynamic State-Dependent Resource Optimization

**DOI:** 10.3389/fneur.2018.00790

**Published:** 2018-10-05

**Authors:** Blerina Latifi, Antoine Adamantidis, Claudio Bassetti, Markus H. Schmidt

**Affiliations:** ^1^Inselspital, Bern University Hospital, University of Bern, Bern, Switzerland; ^2^Department of Neurology, Center for Experimental Neurology, Inselspital, Bern University Hospital, University of Bern, Bern, Switzerland; ^3^Department of Biomedical Research, Inselspital, Bern University Hospital, University of Bern, Bern, Switzerland; ^4^Ohio Sleep Medicine Institute, Dublin, OH, United States

**Keywords:** hypocretin, orexin, melanin concentrating hormone (MCH), sleep function, energy conservation, resource optimization, rapid eye movement (REM) sleep, NREM sleep

## Abstract

The hypocretin (Hcrt) system has been implicated in a wide range of physiological functions from sleep-wake regulation to cardiovascular, behavioral, metabolic, and thermoregulagtory control. These wide-ranging physiological effects have challenged the identification of a parsimonious function for Hcrt. A compelling hypothesis suggests that Hcrt plays a role in the integration of sleep-wake neurophysiology with energy metabolism. For example, Hcrt neurons promote waking and feeding, but are also sensors of energy balance. Loss of Hcrt function leads to an increase in REM sleep propensity, but a potential role for Hcrt linking energy balance with REM sleep expression has not been addressed. Here we examine a potential role for Hcrt and the lateral hypothalamus (LH) in state-dependent resource allocation as a means of optimizing resource utilization and, as a result, energy conservation. We review the energy allocation hypothesis of sleep and how state-dependent metabolic partitioning may contribute toward energy conservation, but with additional examination of how the loss of thermoregulatory function during REM sleep may impact resource optimization. Optimization of energy expenditures at the whole organism level necessitates a top-down network responsible for coordinating metabolic operations in a state-dependent manner across organ systems. In this context, we then specifically examine the potential role of the LH in regulating this output control, including the contribution from both Hcrt and melanin concentrating hormone (MCH) neurons among a diverse LH cell population. We propose that this hypothalamic integration system is responsible for global shifts in state-dependent resource allocations, ultimately promoting resource optimization and an energy conservation function of sleep-wake cycling.

## Introduction

In the 20 years since the discovery of the hypocretin/orexin (Hcrt) system, a growing diversity of physiological and neurobehavioral responses under Hcrt control has challenged identification of a unifying function. A compelling hypothesis suggests that Hcrt plays a role in the integration of sleep-wake neurophysiology with energy metabolism (1–3). Hcrt neurons, for example, are sensors of energy balance through their receptivity to leptin, ghrelin, and glucose (1, 4–6). It has been proposed that the Hcrt system may coordinate the waking response during periods of negative energy balance to promote foraging behavior. However, many questions still remain. Although the loss of Hcrt leads to an increase in REM sleep propensity associated with narcolepsy (7–9), a parsimonious role for Hcrt in both REM sleep regulation and energy metabolism has remained unknown.

Hypocretins 1 and 2 (Hcrt1 and Hcrt2), also called orexins A and B, are excitatory hypothalamic neuropeptides that are derived from a single precursor molecule by proteolytic processing and are largely co-localized in the same neurons (10). They were independently described by two different groups in 1998 (11, 12). While early studies emphasized the role of the Hcrt system in feeding and energy balance, subsequent research focused on sleep-wake regulation based on the discovery that Hcrt dysfunction underlies the sleep disorder narcolepsy.

Hcrt neurons are found exclusively in the posterior lateral hypothalamus (LH) (11–13), numbering between 4,000–5,000 in the rat (13–15) and 50,000–80,000 in humans (16). The anatomy and the projections of the Hcrt system (see below) strongly suggest involvement in diverse physiological functions including sleep-wake control, feeding, thermoregulation, blood pressure, motivation/reward, and neuroendocrine regulation. Long before its discovery, the region of the LH containing Hcrt cells had been implicated in arousal state control (17, 18). It is now well-understood that Hcrt signaling within the LH promotes wakefulness. Indeed, Hcrt neurons are wake-active as measured by Fos expression (19), electrophysiology (20–22), or brain/CSF peptide content (23–25). Hcrt increases arousal when infused into the brain (26–32), and optogenetic stimulation or inhibition of Hcrt signaling increases or decreases wakefulness, respectively (33–36).

Here, on the 20th anniversary of its discovery, we examine a potential role for Hcrt in optimizing efficiencies in energy utilization and, thus, energy conservation. Our recent energy allocation hypothesis of sleep proposes that state-dependent metabolic partitioning at the whole organism level provides greater daily energy conservation through resource optimization than the measured metabolic rate reduction observed during sleep (37, 38). A basic tenet of this hypothesis from an evolutionary perspective is that “optimization of rates of return on energy investments is the singular design principle for the organization of sleep and wakefulness” (p. 126) (37). We propose that optimization of energy expenditures at the whole organism level necessitates a top-down network responsible for coordinating biological operations in a state-dependent manner across organ systems. We will also examine the hypothalamic control of REM sleep vs. wakefulness in the setting of their competing thermoregulatory demands and how loss of thermoregulatory control during REM sleep may enhance resource utilization when REM sleep expression can be modulated as a function of ambient temperature. Finally, we propose that the Hcrt system is part of a dynamic hypothalamic integration system responsible for optimizing global shifts in state-dependent resource allocations central to an energy conservation function of sleep-wake cycling.

## Sleep-wake cycling, resource optimization, and energy conservation

Sleep has long been considered an energy conservation strategy, potentially explaining its universal presence across the animal kingdom (39–41). However, the mechanism by which sleep conserves energy historically has been studied only in terms of the degree to which metabolic rate is reduced during sleep compared to quiet wakefulness, similar in concept to torpor or hibernation. For example, mammals typically decrease metabolic rate by 15–30% during sleep (42–44). For an organism with an 8 h sleep quota per day, this 8 h metabolic rate reduction equates to a calculated energy savings of 5–9% per 24 h. Although significant, this only modest daily energy savings has been considered insufficient to explain the universal presence of sleep, particularly when considering the costs of a sleep strategy, including increased predation risks and lost mating, parental care, and foraging opportunities secondary to the decreased behavioral responsiveness associated with sleep (43, 45, 46).

Moreover, metabolic rate reduction during sleep, analogous to torpor as the principal mechanism of energy conservation, assumes that all biological processes are equally reduced during sleep compared to wake. However, sleep is a highly active metabolic state during which many specific biological operations are upregulated, contradicting the notion that sleep, like hibernation, involves the global downregulation of metabolic processes. These functions specifically upregulated during sleep compared to wake include protein biosynthesis, intracellular transport and membrane repair (47, 48), immune function (49), elimination of biological waste or restorative processes (50–52), and neural network reorganization for memory processing (53–57), to name a few.

**Figure 1:**
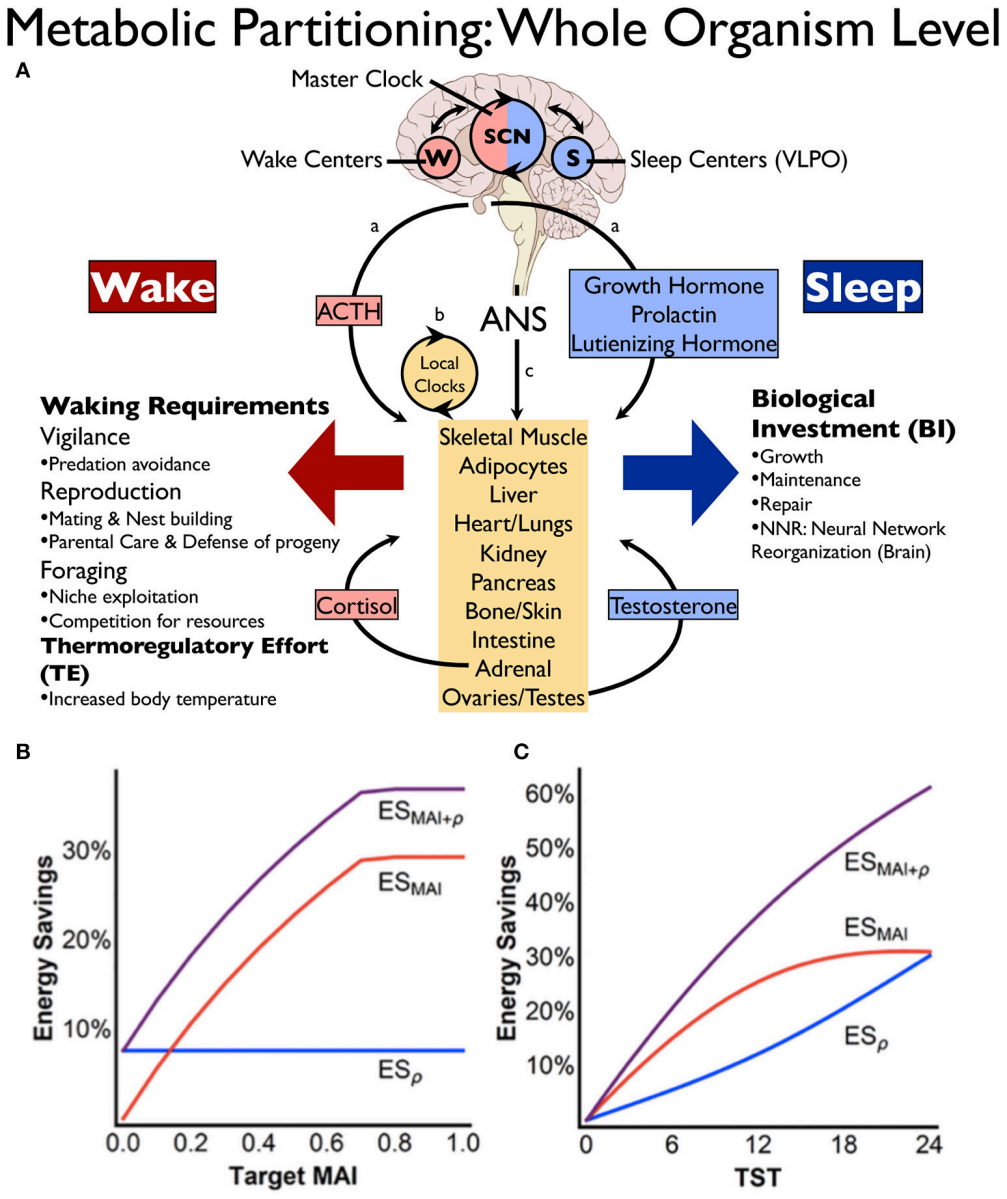
Energy conservation through state-dependent (sleep-wake) metabolic partitioning. **(A)** Control of resource allocations involves both brain and periphery. Within the brain, sleep or wakefulness is expressed based on the interplay between sleep (S) and wake (W) promoting centers, the timing of which is modulated by the suprachiasmatic nucleus (SCN). Wake predominant (red) or sleep predominant (blue) processes in the periphery are synchronized with behavioral state (brain) through: (a), hormonal control, (b) local autonomous circadian clocks in peripheral tissues, and (c) direct descending autonomic nervous system (ANS) innervation from brain to periphery [reprinted with permission from Schmidt (37)]. **(B,C)** Energy savings calculations based on mathematical modeling showing the potential impact of metabolic partitioning **(B)** or sleep quota **(C)** on energy savings derived from sleep-wake cycling. In **(B)**, metabolic partitioning, represented by the metabolic allocation index (MAI), can be varied in the model from 0 to 1 (0 indicating all biological processes are equally performed in both states, whereas MAI = 1 signifies that all functions performed during wake are different from sleep). Target MAI is varied in the figure while holding metabolic rate reduction during sleep (ρ) and total sleep time (TST) constant. Note in the figure that an 8 h sleep quota and a metabolic rate reduction during sleep of 30% (ρ = 0.3) constrain maximum MAI to ~0.7. In **(C)**, TST is varied while holding target MAI = 0.4 and ρ = 0.3. Blue line is energy savings from metabolic rate reduction (ES_ρ_), red line is saving from MAI (ES_MAI_), and purple line is overall energy savings (ES_MAI+ρ_). Reprinted from Schmidt et al. (38).

Our recent energy allocation hypothesis views sleep-wake cycling as a behavioral strategy promoting energy conservation through dynamic state-dependent metabolic partitioning and resource optimization (37, 38). A basic premise of this theory is that the partitioning of metabolic functions by behavioral state occurs at the whole organism level and is not restricted to a single organ or structure. Thus, sleep-wake cycling increases total energy savings through resource optimization beyond what a single organ system could otherwise achieve. Indeed, a great diversity of gene expression is specifically coupled with either sleep or wakefulness in both central and peripheral tissues (47, 48, 58), consistent with state-dependent metabolic partitioning at the whole organism level. As shown in Figure 1A, specific mechanisms are known to synchronize or coordinate brain with periphery according to behavioral state, including state-specific hormonal release (anabolic in sleep, catabolic in wake), synchronization of central and peripheral circadian clocks, and direct autonomic innervation of peripheral tissues (37).

The energy allocation hypothesis of sleep proposes that state-dependent metabolic partitioning provides greater daily energy conservation than the measured metabolic rate reduction observed during sleep (37, 38). Based on relative rates of energy deployment for biological processes upregulated during either wake or sleep, the potential energy savings as derived from state-dependent metabolic partitioning has been calculated using mathematical modeling (38). This modeling suggests that whole organism energy savings is amplified to over 35% per 24 h for an 8 h sleep quota when unique biological processes are partitioned by behavioral state (see Figures 1B,C).

Several important principles emerge from this mathematical modeling, including why sleep may be a better behavioral strategy than quiet wakefulness with respect to resource optimization and energy savings. This modeling suggests that metabolic partitioning by state is constrained if energy deployment toward waking-related processes is maintained during the rest phase, whereas energy savings are amplified if waking-related allocations during the rest phase are eliminated (38). This mechanism of energy conservation requires a system capable of not only monitoring the energy status of the organism, but also integrating many key physiological variables including core and ambient temperature to provide a coordinated output to a wide range of central and peripheral tissues.

## Hypocretin and its role in diverse functions

We will now review the role of Hcrt on modulating a wide range of physiological responses. Understanding these diverse physiological effects will allow us to explore a potential role for Hcrt in a broader perspective as a network control system promoting resource optimization.

### Hcrt in arousal and sleep-wake transitions

The Hcrt system was first linked to sleep function through the discovery that a significant loss of Hcrt neurons leads to narcolepsy, a condition characterized by excessive daytime sleepiness, cataplexy, sleep paralysis and hypnagogic hallucinations (16, 59). These latter symptoms of narcolepsy, including loss of muscle tone and hallucinations, are thought to be components of REM sleep that inappropriately intrude into wakefulness, consistent with a dysregulation of boundary state control in narcolepsy. In canine narcolepsy, a mutation of the Hcrt 2 receptor leads to these manifestations (60). In rats, Hcrt 1 administration (i.c.v.) increases wakefulness in a dose-dependent manner (30), whereas pharmacological antagonism of the Hcrt receptors results in increased NREM and REM sleep and reduces wakefulness in both animals and humans (61). In squirrel monkeys who show a similar diurnal wake pattern to that of humans, Hcrt levels in the cerebro-spinal fluid peak toward the end of the light (waking) period (25).

Optogenetic activation of Hcrt neurons increases the probability of sleep to wake transitions from either NREM or REM sleep (34) throughout both the light and dark phases but with decreased effectiveness following sleep-deprivation and marked increases in sleep pressure (35). On the other hand, optogenetic inhibition of Hcrt neurons during waking in the rest (light) phase increases NREM sleep, while its inhibition during the active (dark) phase has produced mixed effects (33). In support of its role as a waking system, Hcrt has been shown to interact with several neuronal groups that are known to be wake-promoting, such as histaminergic neurons of the tuberomammilary nucleus and noradrenergic neurons of the locus coeruleus (26, 27, 62). In summary, stimulating Hcrt neurons increases the likelihood of transitions into wakefulness, whereas its inhibition increases the likelihood of transitions into sleep.

The narcoleptic phenotype not only leads to excessive daytime sleepiness, but also to fragmented sleep with many brief arousals (63–65). Such transitions between states have also been demonstrated in Hcrt-knockout (KO) mice, a phenotype showing poor maintenance of wakefulness and fragmented NREM sleep even after 8 h of sleep deprivation (66). These transitions result from behavioral state instability, as a loss of Hcrt may to lead to a breakdown of neural control processes that lower the thresholds to transition between behavioral states, producing the fragmented wakefulness and sleep typical of the narcolepsy condition (66). Saper et al. (67) hypothesize that Hcrt acts as a stabilizer on a “flip-flop switch,” avoiding transitional states by not only “flipping” the organism between wake and sleep but also providing stability between two opposing behavioral states (67). In support of this perspective, narcoleptics easily fall asleep during the day but wake up more often at night while leaving the net time asleep unchanged (68, 69).

### Hcrt and thermoregulation

Thermoregulation is tightly integrated with the sleep-wake cycle in mammals and is an important function of Hcrt. In healthy subjects during sleep, decreased heat production from reduced muscle activity and lower basal metabolism contribute to a body temperature that is regulated at a lower level (70, 71). Additionally, peripheral vasodilatation helps decrease the core body temperature during sleep initiation (72). Sleep-promoting neurons in the preoptic area also activate heat loss mechanisms (71, 73, 74), and a modest fall in body temperature is common to mammalian sleep.

Compared to healthy controls, narcoleptics show lower core body temperatures while awake (75) and higher than normal body temperature during sleep (76, 77). Hcrt deficient mice also show similar body temperature abnormalities (78). Hcrt-KO mice show significantly smaller deviations from peak to trough core body temperatures than wild type mice, demonstrating more frequent small fluctuations and fewer large alterations of core body temperature and energy expenditure (78, 79). It appears that narcolepsy is a condition that does not allow the body temperature and basal metabolic rate to decrease properly during sleep or to rise appropriately during active periods. However, it remains to be determined if these frequent, yet small changes in body temperature amplitude directly result from metabolic alterations secondary to Hcrt cell loss or simply indirectly reflect the instability of sleep/wake states in narcoleptics.

An altered pattern of skin-temperature regulation is also reported in patients suffering from narcolepsy (80). In healthy controls, distal skin temperature shows a rhythm that is the inverse to the core body temperature rhythm (81). During daytime in humans, core body temperature is high and distal skin temperature is relatively low, a combination that correlates with optimal vigilance (82–87). In contrast, core body temperature decreases at night, while distal skin temperature is relatively high, signifying optimal sleep (82–87). Indeed, sleep onset is characterized by a rise in temperature of the distal skin compared to the temperature of the proximal skin (known as the distal-to-proximal gradient) (72).

Narcoleptic subjects during the waking period show higher distal skin temperatures with lower proximal skin temperatures, which may either reflect or contribute to the sleepiness of narcolepsy (80) as this combination is associated with sleep onset in healthy subjects (72). It has also been demonstrated that narcoleptic patients are better able to maintain vigilance when their core temperature is artificially raised or when distal skin temperature is experimentally lowered, thereby simulating the conditions known to be correlated with optimal vigilance (75). Taken together, these data demonstrate an inability to appropriately modulate thermoregulatory control according to behavioral state in narcolepsy. The extent to which this breakdown in thermoregulatory responses may also lead to inefficiencies in resource allocations contributing to the narcolepsy phenotype has yet to be explored.

The Hcrt system also plays an important role in increasing core body temperature by activating brown adipose tissue (BAT) (88, 89), suggesting an important role of Hcrt in responding to a cooling of the ambient temperature by activating both waking and heat production (90). BAT plays an important role in regulating body temperature in mammals. It expresses the protein UCP1 which uncouples ATP production from respiration to liberate energy in the form of heat (90). This UCP1-driven heat dissipation is termed “non-shivering thermogenesis” and allows mammals to generate heat without needing to resort to the higher energy demands of shivering to keep warm in cold conditions (91). Non-shivering thermogenesis occurring in BAT is largely under sympathetic control and can be activated by catecholamines (92, 93). Hcrt modulates sympathetic activity and, through its projections to the raphe pallidus, can increase BAT thermogenesis during arousal (88, 90). By increasing body temperature during periods of wakefulness, the Hcrt system may act to optimize metabolism during periods requiring enhanced performance such as food seeking or reproduction (90). In contrast to waking, evidence suggests that BAT thermogenesis may be state-specifically inhibited or reduced during REM-sleep (94, 95). Although narcoleptic patients appear capable of activating BAT during cold ambient temperature exposure (96), more work is needed to determine if narcoleptics show deficiencies with respect to dynamic BAT modulation and coordination with peripheral vasomotor responses.

### Role of Hcrt in food seeking and reward

Hypocretin is also called orexin because of its role in feeding, as orexin means “appetite” in Greek. In mammals, arousal is reduced after feeding and increased during fasting (1, 97–99), and Hcrt neurons are inhibited following food-intake and are activated during periods of fasting (100). In mice with a genetic ablation of Hcrt neurons, normal responses to fasting, such as increased wakefulness and foraging behaviors, are not observed (1, 4, 101). Finally, Hcrt may play a role in mediating shifts in circadian rhythms in response to changes in the timing of food availability or nutritional status. For example, genetic elimination of Hcrt results in reduced food anticipatory activity and the expected temporal expression of numerous clock genes (101, 102).

Food seeking behavior caries an essential survival function and is a potent reward stimulus. Multiple studies show that Hcrt plays an active role in reward processing for both food and drug-seeking behaviors. Human narcoleptics rarely show stimulant abuse or drug seeking behavior even though they are treated for years with stimulants (103). The first observation of a possible role for hypocretins in addiction showed that these neurons play a role in opiate withdrawal (104). Harris et al. (105) demonstrated a potential implication of Hcrt neurons in reward related phenomena showing the activation of Hcrt neurons after acquiring reward and that C-Fos activation in Hcrt neurons is strongly associated with the expression of conditioned place preference (CPP) for drug or natural food rewards. Additionally, Hcrt-KO mice completely lack CPP for morphine (106). Finally, although its etiology is likely multifactorial in origin, narcoleptic patients have a higher risk of depression (107–109). Taken together, these findings indicate that Hcrt neurons play a role in motivation-driven behavior and are part of a circuitry involved in reward processing (110, 111).

### Hcrt and cardiovascular control

Finally, Hcrt also mediates autonomic and cardiovascular effects associated with sleep and waking. For example, there are several well-known sleep-associated cardiovascular changes. One such change is the decrease in blood pressure that occurs during sleep in a variety of species, a phenomenon generally referred to as “dipping” (112–115). Dipping is thought to be influenced by a change in the activity of the autonomic nervous system, since non-dippers have an increase in sympathetic tone at sleep onset as opposed to the normal reduction in sympathetic tone and concomitant increase in parasympathetic tone that typically occurs during sleep (116, 117). Hcrt plays an important role in regulating blood pressure across behavioral states. For example, the lack of Hcrt in transgenic animals is associated with lower systemic blood pressure and a blunting of the decrease in blood pressure during sleep compared to wake (118, 119). Finally, Hcrt increases sympathetic outflow and has effects on the hypothalamic-pituitary-adrenal (HPA) axis, resulting in an increase in catecholamine release (118, 120, 121). Indeed, optogenetic approaches demonstrate that Hcrt neuronal activity regulates corticosterone release through the HPA axis and resulting in behavioral correlates of stress responses (121). These findings highlight the ability of Hcrt to potentially affect wide ranging peripheral tissues through both autonomic and neuroendocrine effects.

In summary, these data demonstrate that Hcrt plays a role in feeding and sleep-wake modulation, as well as numerous other physiological processes such as thermoregulation, motivation and reward, autonomic and neuroendocrine modulation. We will next review how the Hcrt system, together with other LH cell populations, may integrate these physiological responses with respect to the energy status of the organism. Finally, we will propose a unifying role for Hcrt that includes NREM and REM sleep regulation, particularly NREM-REM cycling, with respect to resource optimization and energy conservation.

## The LH in energy balance, behavioral state transitions and resource optimization

In this section, we will describe how, during fasting when energy resources are low, Hcrt promotes wakefulness and food seeking behavior. In contrast, in the presence of a positive energy balance after feeding, other cell types within the LH, such as melanin concentrating hormone (MCH) neurons, promote sleep. We will also examine how the LH, through reciprocal interactions between heterogeneous neuronal populations, may coordinate global shifts in resource utilization through sleep-wake state transitions. Importantly, these reciprocal interactions within hypothalamic circuits suggest that Hcrt not only mobilizes or promotes a waking response, but actively inhibits sleep-related processes and thus contributes to a state-dependent partitioning of biological operations, a mechanism that we propose is central to an energy conservation function of sleep-wake cycling.

### Anterior vs. posterior hypothalamus and behavioral state modulation

As was originally hypothesized in the 1930's, behavioral state control is, in part, anatomically segregated within the hypothalamus (18, 122). Historically, the anterior hypothalamus has been viewed as a “sleep center” since lesions in this area are known to cause long-lasting insomnia, whereas the posterior hypothalamus has been viewed as a “waking center” since its lesioning causes excessive sleepiness [see Saper et al. (67)]. Although this anterior-posterior classification of “sleep” vs. “waking” centers may be an oversimplification given the role other brain circuits (123–125), many of these historical insights still hold true today.

**Figure 2:**
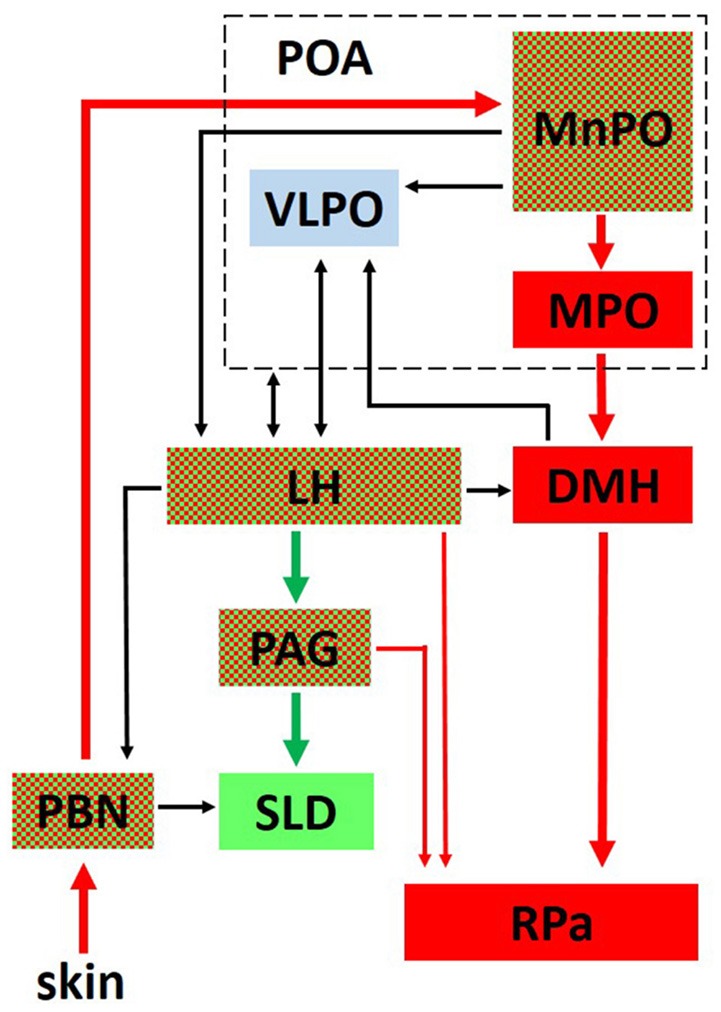
The interplay between critical networks controlling REM sleep (green), thermoregulation (red) or both (checkered). Ambient temperature information from thermosensors in the skin is carried through the dorsal columns of the spinal cord to the parabrachial nucleus (PBN) where it is then relayed to the median preoptic nucleus (MnPO) in the preoptic area (POA). Both warm and cold sensitive neurons in the MnPO relay information to the medial preoptic nucleus (MPO) and to the dorsomedial hypothalamic nucleus (DMH). From there, a major thermoregulatory pathway involves the connection to the raphe pallidus (RPa) which is a major output pathway for the control of brown adipose tissue (BAT). Not shown in the figure is a direct pathway from the MnPO and MPO to the RPa for the output control of vasomotor thermoregulatory responses (137). The LH receives either direct or indirect input from the POA related to sleep and thermoregulatory centers and plays an important role in REM sleep control through its descending connections to the ventrolateral periaqueductal gray (PAG) which projects to the sublaterodorsal tegmental nucleus (SLD) in the pons. Hcrt neurons in the LH send projections to the RPa and play an important role in modulating thermoregulatory control. Reprinted with permission from Cerri et al. (134).

Anteriorly, several structures are particularly noteworthy. The ventrolateral preoptic nucleus (VLPO) within the preoptic area plays an important role in initiating sleep and driving NREM sleep (67, 126, 127). An extensive literature demonstrates that many sleep active neurons within the preoptic area, regions encompassing both the VLPO and the median preoptic nucleus (MnPO), are also warm sensitive and thought to promote sleep when the animal is in a warm, thermoneutral environment (128–132). Although unclear if arising from the same neuronal population, the MnPO also contains temperature sensitive neurons that receive either warm or cold ambient temperature input from the periphery (skin) via relay connections through the brainstem parabrachial nucleus (128, 133) (see Figure 2). Thermoregulatory information from the MnPO and Medial Preoptic nucleus (MPO) are then relayed to the raphe pallidus (RPa), either directly or via the dorsomedial hypothalamus (DMH), for output effector modulation of vasomotor responses and BAT (133–138) (Figure 2). Numerous structures involved in REM sleep regulation, such as the LH and periaqueductal gray (PAG), also control thermoregulatory responses (Figure 2), demonstrating a close anatomical relationship between sleep and thermoregulatory systems.

Initially considered as only a waking center, the posterior LH has been shown to regulate behavioral state transitions from NREM sleep to either REM sleep or wakefulness (1, 139). Although we will focus on the Hcrt and MCH systems, it is important to note that the LH also contains many other cell types involved in sleep, wakefulness and metabolism (3). These include several subtypes of non-MCH GABAergic neurons, including those that trigger wakefulness through inhibition of the thalamic reticular nucleus (140), and others that may drive REM sleep (141, 142) or feeding behavior (143, 144). Finally, the LH receives widespread inputs, and appears to integrate this diverse information for output behavioral state modulation. For example, a prevailing view suggests that information flow from the VLPO and MnPO related to sleep pressure and temperature may either directly or indirectly modulate the LH (134, 136) (see Figure 2).

### Hcrt and MCH in state control and energy balance

In contrast to Hcrt and its predominant role in wakefulness, it is now well-established that the MCH neuronal system modulates REM sleep expression (145–147), at least in part through a GABAergic inhibitory system (148–150). C-Fos studies have shown that MCH neurons are most active after REM sleep hypersomnia (151, 152), consistent with the observation that MCH neurons fire maximally during REM sleep (153). Measurement of MCH levels within the amygdala across the sleep-wake cycle in humans also show that the onset of sleep is associated with an increase in MCH, consistent with a hypothesis that MCH may also play a role in generating NREM sleep (154, 155). Concordantly, administration of an MCH antagonist decreases the amount of REM and NREM sleep and can lengthen the NREM-REM cycle (156). Optogenetic activation of MCH neurons during NREM sleep increases the number of NREM to REM sleep transitions, whereas state-specific activation of MCH neurons during REM sleep prolongs REM sleep bout durations (148).

The Hcrt and MCH neuronal systems are intermingled within the LH and generally fire reciprocally (153, 157). For example, in a head-restrained rat preparation, MCH neuronal activity is maximal during REM sleep when Hcrt neurons are virtually silent. During waking, in contrast, Hcrt neurons are maximally active, while MCH neuronal activity is absent. It should be noted, however, that calcium imaging of MCH neurons shows an increase in activity during some waking behaviors specifically related to novel object presentation (158). Although the relationship between MCH and Hcrt appears complex, evidence suggests either a direct or indirect reciprocal inhibition (159). Optogenetic stimulation of Hcrt neurons, for example, promotes inhibition of some MCH neurons by increasing presumably GABAergic interneuronal input onto MCH cells, whereas other MCH neurons are directly excited by Hcrt (159). The colocalization of endogenous opioids in Hcrt neurons, in contrast, appear to hyperpolarize MCH cells (160–162). Whatever the mechanism, the interconnection between Hcrt and MCH generally favors a reciprocal firing pattern of these two neuronal systems.

Hcrt and MCH neurons also generally respond with opposite activity patterns to the nutritional state of the organism, and thus energy balance, through their receptivity to numerous biomarkers related to energy status. To illustrate, Hcrt neurons are inhibited by biomarkers which are released in response to food intake, such as leptin (satiety hormone produced by adipose tissues), glucose, and neuropeptide Y (1, 4, 5, 163–165), whereas ghrelin, which is an appetite-stimulating hormone produced in the gut, increases Hcrt activity (6). The inhibitory response of Hcrt neurons mediated by leptin appears secondary to GABAergic interneurons within the LH colocalizing neurotensin and expressing the leptin (LepRb) receptor (166–169). Finally, Hcrt neurons express the adenosine A1 receptor and are inhibited by extracellular adenosine (170–172), suggesting that adenosine can potentially promote sleep by decreasing Hcrt activity.

MCH neurons are responsive to insulin and glucose, but they demonstrate an opposite response pattern compared to Hcrt neurons (173, 174). For example, approximately 70% of MCH neurons are excited by physiological elevations in glucose (5 mM) and dose-dependently hyperpolarize as glucose levels are lowered, whereas Hcrt neuronal activity shows opposite effects to these same glucose concentrations (174, 175). This coordinated response from glucose involving inhibition of Hcrt with activation of MCH appears to promote sleep, thus activating sleep-dependent processes when body energy resources are high after feeding (1, 174). Finally, MCH neurons also release Nesfatin-1, a satiety hormone, and disruption of Nesfatin-1 signaling decreases REM sleep expression (176).

Taken together, these data demonstrate multiple complimentary and redundant mechanisms by which the LH may tightly monitor energy status of the organism. Moreover, these data are consistent with the hypothesis that the posterior LH integrates energy status and sleep-wake modulation, at least in part through the reciprocal activation of the MCH and Hcrt systems.

### MCH and feeding behavior

Although MCH promotes sleep and is responsive to energy status, a considerable body of literature indicates that MCH, like Hcrt, also regulates feeding behavior. Interestingly, the MCH system may promote feeding behavior while also decreasing both locomotor activity and BAT thermogenesis, thereby contributing to a positive energy balance and potentially conserving energy for homeostatic purposes (177–187). Diniz and Bittencourt (188) suggest that MCH can drive periodic feeding, even when energy balance is positive, given the potential danger in waiting for energy levels to deplete. Indeed, when energy balance is positive and food is available, MCH may promote feeding, thereby maintaining a positive energy-balance. If food is not available, but energy balance is still favorable, MCH may promote a change of state, favoring sleep with a concomitant decrease in both locomotor activity and thermoregulatory defenses (188). Diminishment of energy reserves eventually leads to decreased glucose levels, causing decreased excitability of MCH cells and, together with increased Ghrelin, an excitation of Hcrt activity (174). Hcrt activity, therefore, may predominate when the animal reaches a negative energy balance, leading to increased wakefulness and thermogenesis, both necessary conditions for active food seeking.

### Hcrt and MCH as network control systems

**Figure 3:**
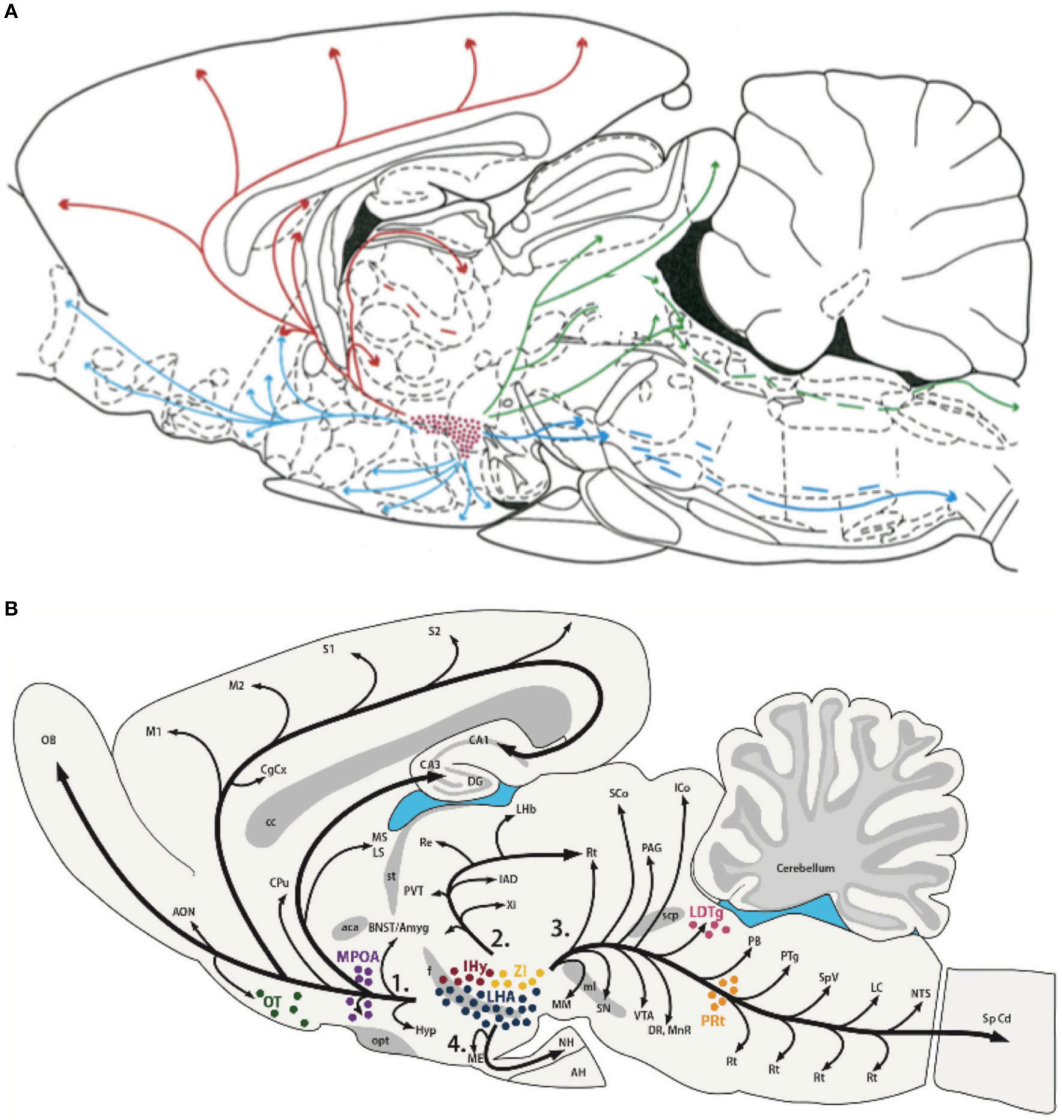
Efferent projections of the hypocretin/orexin (Hcrt) **(A)** and melanin concentrating hormone (MCH) **(B)** systems. The extensive efferent projections from both the Hcrt and MCH neuronal populations originating from within the LH generally overlap and include similar target structures. The diversity of their projections, together with their reciprocal activity patterns and known opposing effects on target systems, is consistent with their hypothesized role in modulating global shifts in network activity and coordinating behavioral state transitions. Figures reprinted with permission **(A)** Peyron et al. (13), and **(B)** Diniz and Bittencourt (188). For abbreviations in **(B)**, see Diniz and Bittencourt (188).

Consistent with their roles in behavioral state modulation and global shifts in peripheral responses, both Hcrt and MCH neuronal populations show wide-spread projections throughout the brain to similar brain areas as shown in Figure 3 (13, 189–191). Peyron et al. (13) observed that Hcrt neurons project throughout the hypothalamus and to extrahypothalamic areas, the densest of which involving the locus coeruleus. Fibers are also observed in the septal nuclei, bed nucleus of the stria terminalis, paraventricular and reuniens nuclei of the thalamus, zona incerta, subthalamic nucleus, central gray, substantia nigra, raphe nuclei, parabrachial area, medullary reticular formation, and the nucleus of the solitary tract (13). For melanin-concentrating hormone, similar projections are observed (190, 192), suggesting that MCH acts on the same brain structures, but with potentially opposing effects. These widespread projections to diverse brain structures, together with the known impact of the Hcrt and MCH systems on many physiological functions such as food intake, regulation of blood pressure, the neuroendocrine system, body temperature and the sleep-wake cycle, places both Hcrt and MCH in key positions. They can be considered top-down network control systems in behavioral state modification while also controlling physiological responses appropriate for the behavioral state. Moreover, they are in a position within the hypothalamus to receive diverse information related to temperature, energy status and homeostatic sleep pressure, suggesting that these opposing neuronal systems are part of a complex integration system to regulate output control for behavioral state and its physiological correlates.

## To REM or to wake? optimizing resource allocations within sleep

After entering NREM sleep, an organism must make a decision as to when it should transition back to wake or, instead, enter REM sleep. One of the most consistent findings in mammalian sleep is the highly structured cycling between NREM and REM sleep. Although the purpose of this cycling is not understood, the duration from one REM period to the next is markedly consistent within any given species of mammal even when considering inter-individual variation. Indeed, one of the strongest phylogenetic correlations across species with respect to sleep parameters thus far examined is between REM-REM cycle length and body mass (193–196). That is, species with a smaller body mass, such as a mouse, cycle much faster from one REM period to another and exhibit much shorter REM sleep bout durations compared to species with a larger body mass such as in man or the elephant. However, the slope of this positive correlation between body mass and cycle length is considerably lower than what one would expect if this correlation were simply based on metabolic rate [see (37, 195)].

We recently proposed that REM sleep cycles with NREM sleep based on thermoregulatory constraints as a means of optimizing REM sleep expression and resource allocations (37). During REM sleep, thermoregulatory control is suspended (197, 198). Although mammals will pant, shiver or sweat during NREM sleep if thermally challenged, these thermoregulatory defenses cease during REM sleep, even when sleeping in ambient temperatures well outside of the thermoneutral zone (TNZ). Thermoregulation and the ability to maintain a constant body temperature is the most energetically expensive biological function encountered by endotherms, increasing energy requirements approximately 10-fold over a similar sized ectotherm (199–201). A constraint of REM sleep is that the longer any single bout of this sleep state, the more likely core body temperature may deviate toward the ambient temperature (202, 203). As a result, REM sleep bout durations are constrained in large part by the animal's surface area to volume ratio (its ability to retain heat) if the animal is to avoid spending excess energy to defend the core temperature because of REM sleep (37).

The energy allocation hypothesis postulates that REM sleep is a behavioral strategy that reallocates resources away from costly thermoregulatory defenses into REM sleep-specific biological functions to improve reproductive success or evolutionary fitness (37). Indeed, although REM sleep is a time of generalized skeletal muscle atonia associated with decreases in BAT, core and liver temperatures (95), brain temperature and brain metabolic rate markedly increase during REM sleep (95, 204, 205). Even though brain metabolic rate significantly increases during REM sleep, whole organism metabolic rate remains unchanged when comparing NREM to REM sleep (42, 79, 206–209). This finding suggests a major internal reallocation of resources during transitions from NREM to REM sleep while maintaining sleep as a net energy neutral state with respect to whole organism energy consumption. The central nervous system and the reproductive system (as seen by penile erections during REM sleep) appear to be major beneficiaries of this energy allocation strategy of REM sleep (37). The cost of this strategy is that the animal is more susceptible to thermal challenges. However, if the organism can cycle NREM and REM sleep in a manner that optimizes total REM sleep quantity while minimizing the need for thermoregulatory defense, such a strategy would by highly advantageous if it achieves greater CNS benefit at a lower, whole organism, energy cost (37).

It is well-established that ambient temperature warming toward the high end of the TNZ increases REM sleep expression across mammalian species, whereas any deviation away from thermoneutrality preferentially decreases REM sleep over NREM sleep (202, 210–215). Moreover, increases in ambient temperature, even within the narrow TNZ, increase NREM to REM sleep transitions, increase REM sleep bout durations, and shorten REM-to-REM sleep cycle lengths (210, 212, 216).

**Figure 4:**
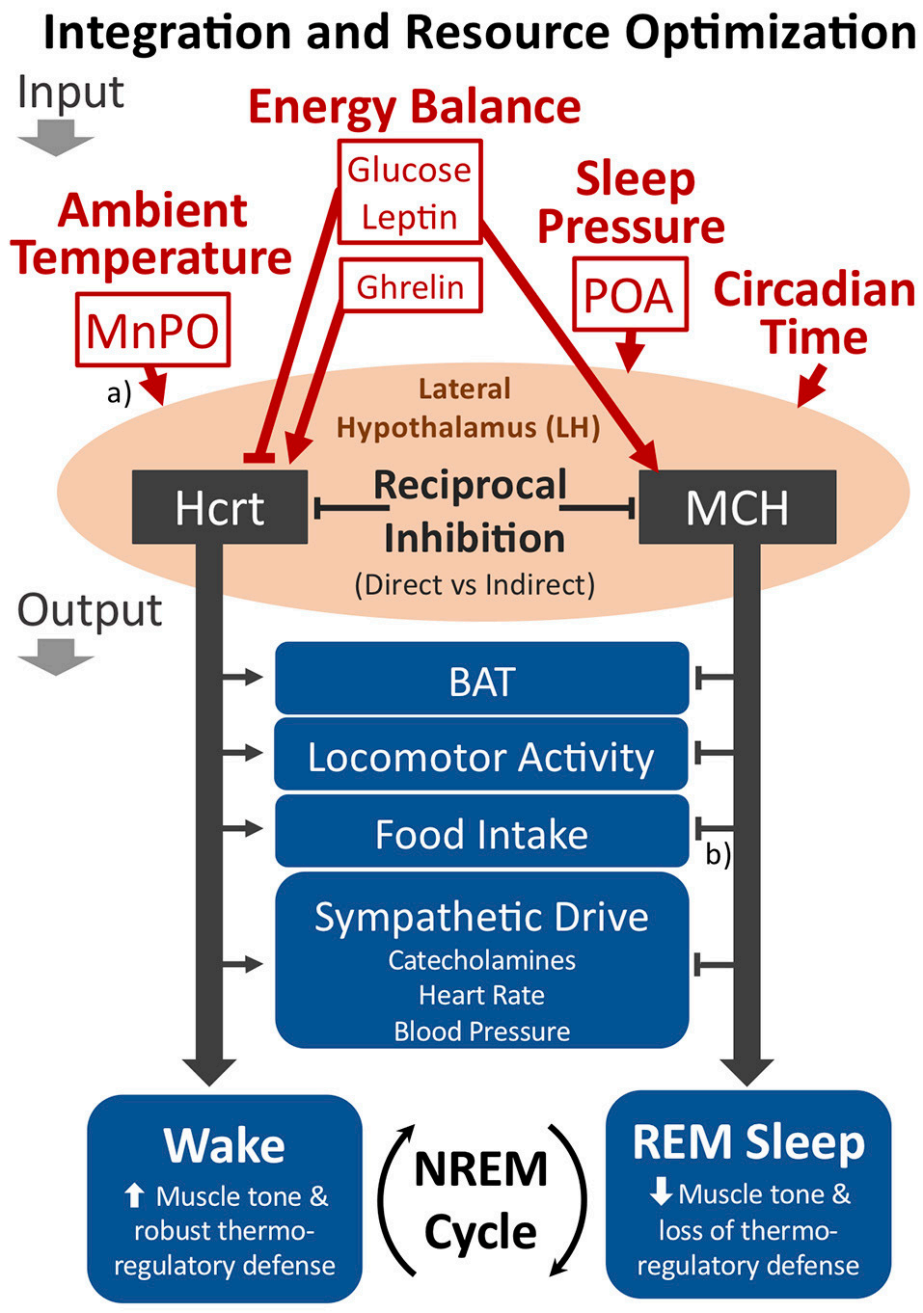
The proposed role of the LH as an integrator of key inputs related to ambient temperature, energy balance, and sleep pressure for the cycling of NREM sleep to either wakefulness or REM sleep. The Hcrt and MCH neuronal systems within the LH show reciprocal firing patterns, suggesting either direct or indirect reciprocal inhibition. If Hcrt activity predominates, the behavioral state of wakefulness is favored, including the promotion of brown adipose tissue (BAT) activation, locomotor activity, food intake, and sympathetic drive. With MCH activity predominating, however, REM sleep is favored. The LH may optimize resource allocations within sleep by modulating the cycling of NREM sleep to either REM sleep or wakefulness (see text). Additional relevant inputs to the LH not depicted in the figure include information pertaining to positive or negative emotional content. (a) The MnPO and POA contain warm sensitive sleep active neurons that may directly innervate the LH. Additionally, the MnPO contains thermosensitive neurons receiving input from the periphery that may also indirectly convey thermoregulatory information to the LH. (b) Although the MCH system appears to have opposing output effects with respect to Hcrt, MCH may either increase or decrease food intake depending on food availability and energy status (see text). Straight arrowheads or bars represent activation or inhibition, respectively. Not shown in the figure are additional cell types within the LH that are involved in behavioral state transitions, such as GABAergic neurons that project to the thalamic reticular nucleus for activation of wakefulness (140). MnPO, median preoptic nucleus; POA, preoptic area.

The LH may play an important role in integrating inputs related to ambient temperature, energy balance and sleep pressure for the output control of transitions from NREM sleep to either wakefulness or REM sleep (see Figure 4). As shown in Figures 2 and 4, the LH receives either direct or indirect inputs regarding ambient temperature and sleep drive from the preoptic area (134). Moreover, the Hcrt and MCH systems are both receptive to the energy status of the organism as described above. The reciprocal firing patterns of the Hcrt and MCH systems, together with their opposing effects on diverse peripheral tissues and behavioral state (REM vs. wake), implicate these two neuronal groups within the LH as critical for its output control. In this view, the MCH system will be favored during warm thermoneutral ambient temperatures, particularly when energy balance is positive or during high sleep pressure, thus increasing the probability of transitions from NREM to REM sleep, decreasing the REM-to-REM sleep cycle length, or increasing REM sleep bout durations. We predict that disruption to either the Hcrt or MCH systems will adversely affect normal output control of NREM sleep transitions with respect to the appropriate integration of such sensory inputs.

Consistent with the energy allocation hypothesis, we propose that the cycling of NREM and REM sleep optimizes resource allocations within sleep by integrating the competing benefits from either thermoregulation vs. activation of REM sleep-coupled functions, including memory consolidation (217, 218), sensory-motor integration (219), visual system development and maintenance (220–222), and reproductive function (37, 223). Organisms that optimize such competing investments at lower, whole organism, energy costs would carry a selective advantage, particularly if able to opportunistically increase REM sleep in warm, thermoneutral, ambient temperatures, or selectively reduce REM sleep if ambient temperatures deviate from thermoneutrality. The LH and the Hcrt system appear to play a major role in optimizing behavioral state transitions. Indeed, loss of Hcrt, as seen in narcolepsy, leads to the abnormally frequent brief awakenings during sleep with behavioral state instability and the inappropriate intrusion of REM sleep or its associated components into wakefulness.

## Summary

In the 20 years since the discovery of Hcrt, our field has come to appreciate the great diversity of physiological processes under its control, including sleep-wake modulation, feeding behavior, thermoregulation, reward processing, sympathetic output, and cardiovascular control. A compelling hypothesis has been that the Hcrt system integrates the sleep-wake cycle with energy status of the organism given its receptivity to signals of energy balance. Although Hcrt may promote wakefulness and feeding behavior during periods of hunger, a parsimonious role for Hcrt in REM sleep regulation with respect to energy or energy balance has yet to be addressed.

We propose that Hcrt and MCH, through their complex interactions with heterogeneous neuronal populations within the LH, dynamically optimize energy utilization through global shifts in resource allocations. These global shifts are achieved through sleep-wake transitions and the coupling of specific biological processes with behavioral state (37, 38). Mathematical modeling suggests that energy savings from sleep-wake cycling are amplified as either waking-related allocations are eliminated during sleep or sleep-dependent functions are decreased during wakefulness (38). Moreover, this modeling shows that metabolic partitioning, or resource optimization, involving both central and peripheral tissues has a greater theoretical impact on total daily energy conservation than what a single organ or structure could otherwise achieve. Optimization of resource allocations at the whole organism level requires a top-down network control system capable of integrating key input variables such as energy status, thermoregulatory demands, and homeostatic sleep need. The Hcrt and MCH systems through their diverse hypothalamic inputs and their extensive efferent projections are ideal candidate structures for this role.

We further postulate that optimization of resource allocations not only occurs between wake and sleep, but also within sleep through NREM-REM sleep cycling. Through an integration of thermoregulatory input, Hcrt and other hypothalamic circuits such as the MCH system appear to modulate the probability of behavioral state transitions from NREM sleep to either REM sleep or wakefulness. If the circumstances are not ideal for REM sleep, such as when ambient temperatures deviate from thermoneutrality and the organism must resort to thermoregulatory defenses such as shivering or BAT thermogenesis, Hcrt will inhibit REM sleep by promoting arousal and heat production. However, if the ambient temperature is within thermoneutrality, the organism may forego thermoregulatory defense and, instead, opportunistically invest into REM sleep coupled biological processes.

Future research is also needed to better understand mechanisms, including costs and benefits, of dynamic resource optimization. For example, although total (24-h) energy expenditure is decreased in Hcrt-KO mice compared to WT controls (79), the loss of Hcrt carries significant costs with respect to decreased locomotor activity, feeding behavior, or reward processing, as well as disruption of thermoregulatory control, and an inability to sustain prolonged waking periods. Organisms must expend energy not only to obtain energy, but also to maximize reproductive success and evolutionary fitness. We advance the hypothesis that the LH promotes optimization of such expenditures. This optimization brings efficiencies that ultimately allow organisms to essentially achieve more with less. This theoretical construct is consistent with the energy allocation hypothesis of sleep function (37, 38), providing a parsimonious perspective on the role of the LH in the cycling of NREM sleep, REM sleep and wakefulness as a means of dynamically optimizing resource allocations and the promotion of energy conservation.

## Author contributions

BL has written the majority of the text. AA and CB supported this article by revising it critically. MHS is responsible for the concepts and design of the review, as well as writing significant passages and text editing.

### Conflict of interest statement

The authors declare that the research was conducted in the absence of any commercial or financial relationships that could be construed as a potential conflict of interest.
